# Associations among cardiovascular and cerebrovascular diseases: Analysis of the nationwide claims-based JROAD-DPC dataset

**DOI:** 10.1371/journal.pone.0264390

**Published:** 2022-03-11

**Authors:** Michikazu Nakai, Yoshitaka Iwanaga, Yoko Sumita, Shinichi Wada, Haruhiko Hiramatsu, Koji Iihara, Takahide Kohro, Issei Komuro, Tomohiro Kuroda, Tetsuya Matoba, Masaharu Nakayama, Kunihiro Nishimura, Teruo Noguchi, Tadamasa Takemura, Teiji Tominaga, Kazunori Toyoda, Kenichi Tsujita, Satoshi Yasuda, Yoshihiro Miyamoto, Hisao Ogawa

**Affiliations:** 1 National Cerebral and Cardiovascular Center, Suita, Japan; 2 Department of Medical Informatics, Jichi Medical University Hospital, Shimotsuke, Japan; 3 Department of Cardiovascular Medicine, Graduate School of Medicine, The University of Tokyo, Tokyo, Japan; 4 Division of Medical Information Technology and Administration Planning, Kyoto University Hospital, Kyoto, Japan; 5 Department of Cardiovascular Medicine, Kyushu University Graduate School of Medical Sciences, Fukuoka, Japan; 6 Department of Medical Informatics, Tohoku University Graduate School of Medicine, Sendai, Japan; 7 Graduate School of Applied Informatics, University of Hyogo, Kobe, Japan; 8 Department of Neurosurgery, Tohoku University Hospital, Tohoku University Graduate School of Medicine, Sendai, Japan; 9 Department of Cardiovascular Medicine, Graduate School of Medical Sciences, Kumamoto University, Kumamoto, Japan; 10 Department of Cardiovascular Medicine, Tohoku University Hospital, Tohoku University Graduate School of Medicine, Sendai, Japan; 11 President, Kumamoto University, Kumamoto, Japan; Faculdade de Medicina de São José do Rio Preto, BRAZIL

## Abstract

Cardiovascular and cerebrovascular diseases are frequently interconnected due to underlying pathology involving atherosclerosis and thromboembolism. The aim of this study was to investigate the impact of clinical interactions among cardiovascular and cerebrovascular diseases on patient outcomes using a large-scale nationwide claims-based dataset. Cardiovascular diseases were defined as myocardial infarction, heart failure, atrial fibrillation, and aortic dissection. Cerebrovascular diseases were defined as cerebral infarction, intracerebral hemorrhage, and subarachnoid hemorrhage. This retrospective study included 2,736,986 inpatient records (1,800,255 patients) at 911 hospitals from 2015 to 2016 from Japanese registry of all cardiac and vascular disease-diagnostic procedure combination dataset. Interactions among comorbidities and complications, rehospitalization, and clinical outcomes including in-hospital mortality were investigated. Among hospitalization records that involved cardiovascular disease, 5.9% (32,686 records) had cerebrovascular disease as a comorbidity and 2.1% (11,362 records) included an incident cerebrovascular complication after hospitalization. Cerebrovascular disease as a comorbidity or complication was associated with higher in-hospital mortality than no cerebrovascular disease (adjusted odds ratio (OR) [95% confidence interval]: 1.10 [1.06–1.14], 2.02 [1.91–2.13], respectively). Among 367,904 hospitalization records that involved cerebrovascular disease, 17.7% (63,647 records) had cardiovascular disease listed as comorbidity and 3.3% (11,834 records) as a complication. Only cardiovascular disease as a complication was associated with higher in-hospital mortality (adjusted OR [95% confidence interval]: 1.29 [1.22–1.37]). In addition, in-hospital mortality during rehospitalization due to the other disease was significantly higher than mortality during the hospitalization due to the first disease. In conclusion, substantial associations were observed between cardiovascular and cerebrovascular disease in a large-scale nationwide claims-based dataset; these associations had a significant impact on clinical outcomes. More intensive prevention and management of cardiovascular and cerebrovascular disease might be crucial.

## Introduction

According to the World Health Organization, cardiovascular and cerebrovascular diseases are the most frequent causes of death worldwide, accounting for 31% of all deaths in 2016 globally [1]. Both are frequently interconnected with common risk factors and underlying pathology such as atherosclerosis, a condition that damages medium and large arteries. In addition, cardiovascular risk factors are associated with the development of arrhythmias such as atrial fibrillation (AF), which predisposes a person to cerebrovascular events when blood clots in the atria and ventricles embolize to the brain. Thus, cardiovascular disease is a frequent cause of cerebrovascular compromise, and vice versa. For example, in older reports, approximately 2–6% of patients died from cardiac causes within 3 months of ischemic stroke [2, 3]. Ischemic stroke is a feared complication of acute myocardial infarction (MI), which is also a strong predictor of mortality [4]. Several cardiac disorders such as AF, valvular disease, and acute MI, are associated with an increased risk of ischemic stroke. Therefore, it is important to acknowledge not only the characteristics of each disease, but also the interrelationships among these diseases. Cardiovascular-cerebrovascular disorders require a multidisciplinary diagnostic and therapeutic approach [5].

Even though most previous studies have recognized the interaction among these diseases, they typically analyzed a dataset from a registry or cohort, which limits the sample size and the number of participating institutions [6, 7]. A comprehensive understanding of the associations among cardiovascular and cerebrovascular diseases and how they manifest as outcomes in clinical practice remains unclear in this era of big data. The aim of this study was to investigate the impact of clinical interactions among cardiovascular and cerebrovascular diseases on patient outcomes using a large-scale nationwide claims-based Japanese Registry Of All cardiac and vascular Disease-Diagnostic Procedure Combination (JROAD-DPC) dataset. First, clinical relationships and outcomes were investigated in terms of comorbidities and complications in hospitalized patients. Second, in-hospital mortality was assessed in the rehospitalization analysis of cardiovascular and cerebrovascular diseases.

## Methods

### Data source

The Diagnosis Procedure Combination (DPC), which is maintained by the Ministry of Health, Labour and Welfare of Japan, is a patient classification method for inpatients in the acute phase of illness. It has been developed as an assessment tool intended to make acute inpatient care transparent and to standardize, evaluate, and improve the quality of Japanese medical care. In the DPC dataset, all procedures and prescriptions during hospitalization are recorded according to the Japanese fee schedule for reimbursement [8, 9]. The Japanese Circulation Society has developed a nationwide claim database, JROAD-DPC, using data from the Japanese DPC, which includes a unique hospital identifier, age, sex, main diagnosis, comorbidities, length of hospital stay (LOS), in-hospital medications, in-hospital cost, and discharge status. This dataset extracts only records that contain cardiovascular or cerebrovascular diseases in any diagnosis category. Of all eligible DPC hospital, 75.8% and 79.2% had 300–500 beds in 2015 and 2016, respectively. Of all eligible DPC hospitals with more than 500 beds in Japan, the JROAD-DPC dataset covers 83.6% of hospitals in 2015 and 93.2% in 2016 [10]. In 2016, 812 hospitals submitted their DPC datasets to the JROAD-DPC database, which covered 68% of hospital beds in all DPC hospitals in Japan. Validation studies for this database have also been conducted previously; details have been described elsewhere [8, 11].

### Diagnosis

Cardiovascular diseases were defined as MI, heart failure (HF), AF, and aortic dissection (AD). The International Classification of Diseases (ICD)-10 diagnosis codes were I21, I22, I24 for MI, I50 for HF, I48 for AF, and I71.0 for AD. Cerebrovascular diseases were defined as cerebral infarction (CI), intracerebral hemorrhage (ICH), and subarachnoid hemorrhage (SAH). ICD-10 codes were I63 for CI, I61 for ICH, and I60 for SAH.

Identification of patients hospitalized with cardiovascular or cerebrovascular disease was based on the index ICD-10 code in any of the following four data fields: main diagnosis, admission-precipitating diagnosis, most resource-consuming diagnosis, and second most resource-consuming diagnosis. Comorbidity was defined as the presence of the index ICD-10 code in the diagnostic category of comorbidity. Complication was defined as the presence of the index ICD-10 code in the field for main diagnosis, admission-precipitating diagnosis, most resource-consuming diagnosis, second most resource-consuming diagnosis, or diagnostic category of complication.

### Study population

**Fig 1:**
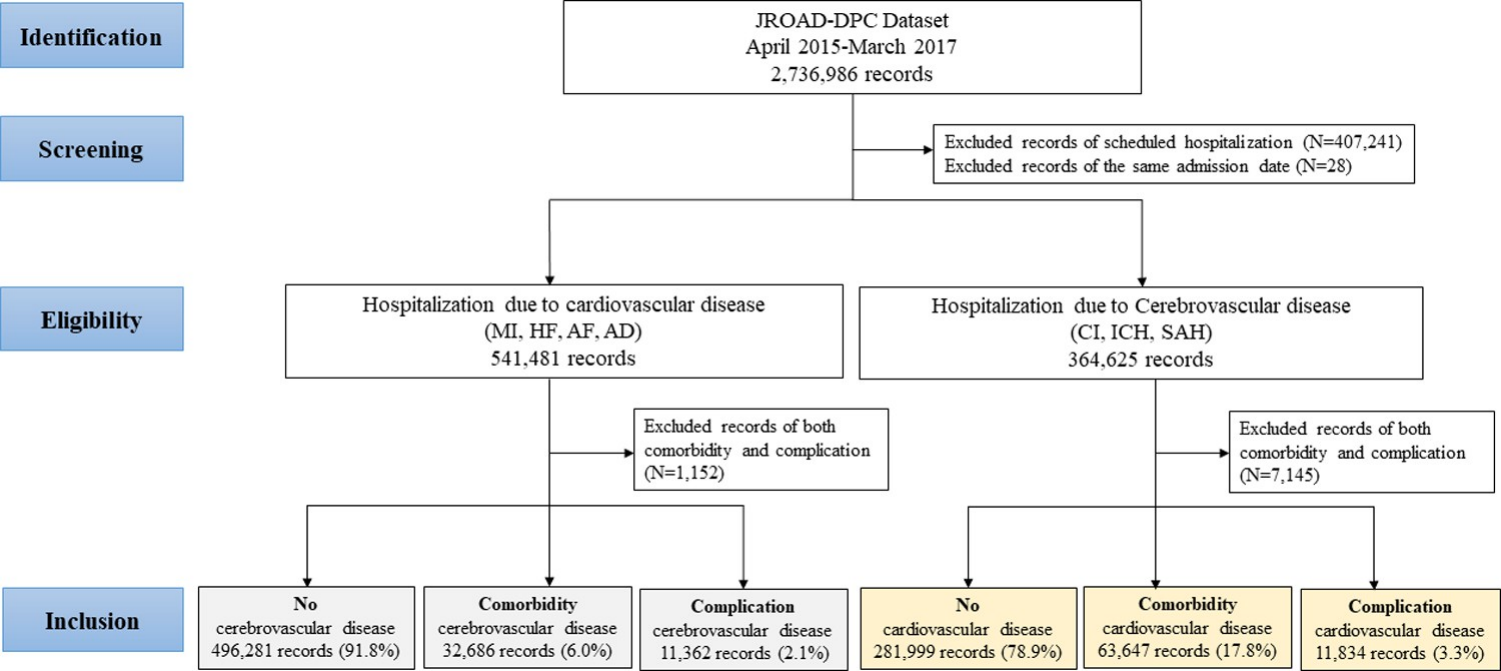
Study flow charts for comorbidities and complications of cardiovascular and cerebrovascular diseases in JROAD-DPC. AD, aortic dissection; AF, atrial fibrillation; CI, cerebral infarction; HF, heart failure; ICH, intracerebral hemorrhage; JROAD-DPC, Japanese Registry Of All cardiac and vascular Disease-Diagnostic Procedure Combination; MI, myocardial infarction; SAH, subarachnoid hemorrhage.

Data in the JROAD-DPC dataset from April 1, 2015 to March 31, 2017 were analyzed in this study. Records for scheduled hospitalizations and multiple admission on the same date were excluded. In addition, records with a disease listed as both a comorbidity and a complication were excluded from the final analysis (Fig 1).

### Rehospitalization analysis

When a patient is first hospitalized due to cardiovascular disease and rehospitalized due to cerebrovascular disease, two scenarios can be considered:

- The cardiovascular disease can be a comorbidity and hospitalization due to cerebrovascular disease appeared in the first record
- Hospitalization due to cardiovascular disease can occur without prior cardiovascular disease as a comorbidity in the first record and rehospitalization due to cerebrovascular disease appeared in the second record.

**Fig 2:**
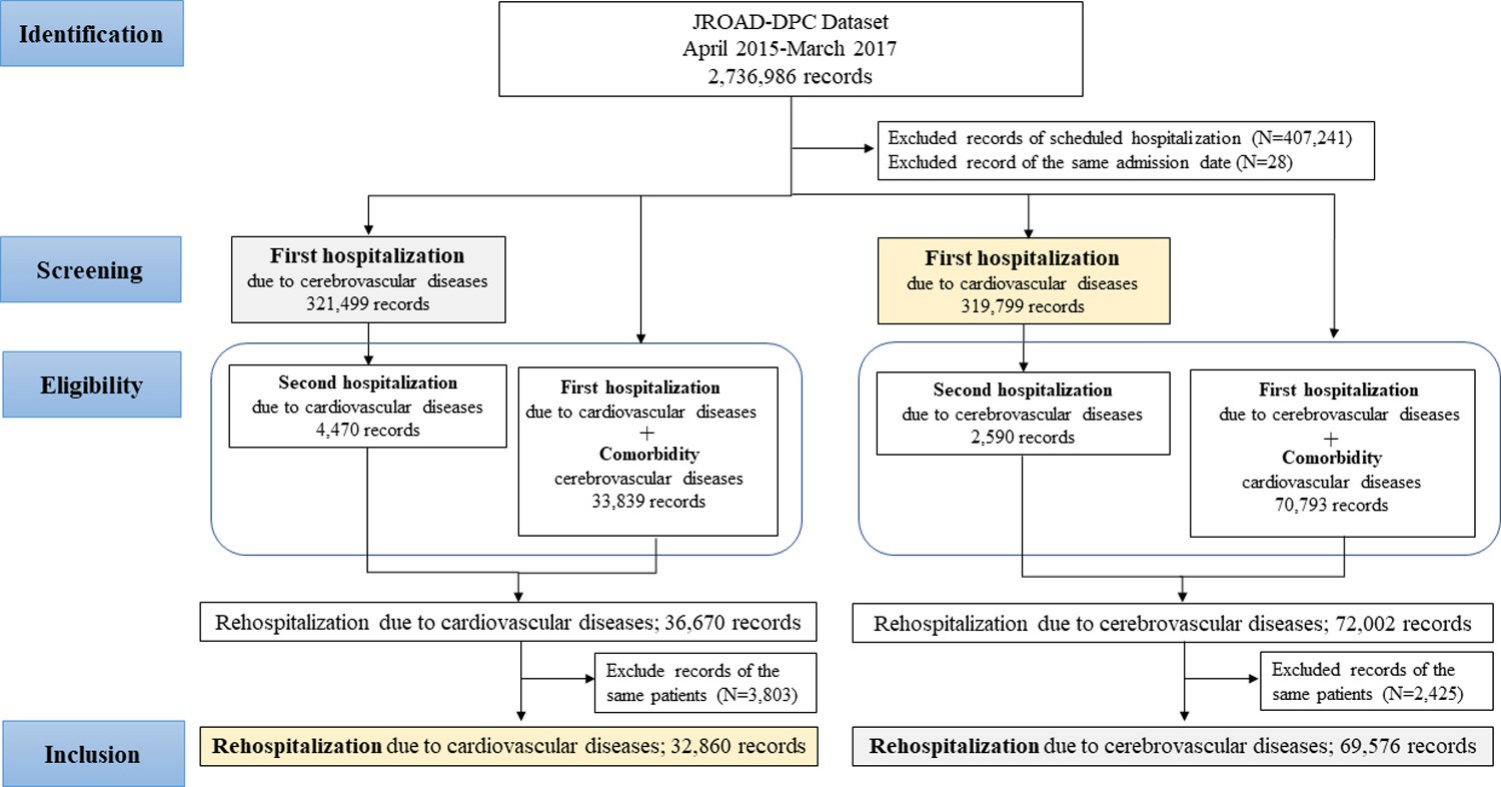
Study flow charts showing analyzes of rehospitalization for cardiovascular and cerebrovascular diseases. AD, aortic dissection; AF, atrial fibrillation; CI, cerebral infarction; HF, heart failure; ICH, intracerebral hemorrhage; JROAD-DPC, Japanese Registry Of All cardiac and vascular Disease-Diagnostic Procedure Combination; MI, myocardial infarction; SAH, subarachnoid hemorrhage.

A similar pattern can be adapted for the first hospitalization due to cerebrovascular disease and rehospitalization due to cardiovascular disease (Fig 2). In the comparison of mortality, the first hospitalization was defined as the first hospitalization due to cardiovascular or cerebrovascular disease without any comorbidities of the other disease during the study period.

### Ethics statement

The study was conducted according to the 1964 Declaration of Helsinki and its later amendments. It was approved by the National Cerebral and Cardiovascular Center ethics committee (authorization number, M29-024-2).

### Statistical analysis

Data are expressed as medians (inter-quartile range) for continuous variables. Categorical data are expressed as numbers (%). Two-sample tests of proportions were conducted. There were missing data for age in 10,065 (0.37%) records and for in-hospital costs for 15,357 (0.56%) records. The median value for these variables was calculated with the complete dataset. Univariate and multivariable multilevel mixed effects logistic regression models with institution as a random intercept were performed to estimate odds ratios (ORs) and 95% confidence intervals for in-hospital mortality. In order to improve the efficiency of the analysis, basic demographic variables were included in Model I and comorbidities that are strongly related to cardiovascular and cerebrovascular diseases were further included in Model II. Multivariable analysis was conducted with two models. Model I adjusted for age and sex. Model II adjusted for age, sex, and comorbidities (hypertension, diabetes mellitus, hyperlipidemia, and chronic kidney disease). In addition, all hospital costs and charges were converted into US dollars according to the current exchange rate (1 US dollar = 106.00 yen). All statistical analyses were conducted using SAS 9.4 (SAS Institute, Cary, NC, USA) and STATA 16.1 (College station, TX, USA).

## Results

### Cardiovascular and cerebrovascular diseases as comorbidities and complications

Among 2,736,986 records of 1,800,255 patients at 911 hospitals, 541,481 records were extracted as hospitalization due to cardiovascular disease and 364,625 records were extracted as hospitalization due to cerebrovascular disease (Fig 1). Among all records involving cardiovascular hospitalization, cerebrovascular comorbidities were observed in 32,686 (6.0%) records and cerebrovascular complications were observed in 11,362 (2.1%) records. Among all records, involving cerebrovascular hospitalization, cardiovascular comorbidities were observed in 63,647 (17.8%) records and cardiovascular complications were observed in 11,834 (3.3%) records. Patients with cardiovascular disease and cerebrovascular comorbidities or complications were elderly and had worse in-hospital outcomes such as in-hospital mortality, score for activities of daily living (ADL) at discharge, and LOS, than patients with no cerebrovascular disease (Table 1A). These outcomes were worse in patients with cerebrovascular complications than those with cerebrovascular comorbidities and hospitalization costs were higher. Similarly, in patients hospitalized due to cerebrovascular disease, there were significant differences in these outcomes among patients with cardiovascular comorbidities or complications versus no cardiovascular disease (Table 1B). However, these differences were smaller than the differences among patients hospitalized due to cardiovascular disease.

**Table 1:** Patient characteristics and outcomes.

**(A)**				
		**Cardiovascular disease**		
		**No cerebrovascular disease**	**Cerebrovascular comorbidity**	**Cerebrovascular complication**
Number		496,281	32,686	11,362
Age group, years		76 (66–85)	81 (73–87)	79 (69–86)
	<60	70,399 (14.2%)	1,560 (4.8%)	1,080 (9.6%)
	60–69	95,138 (19.2%)	4,190 (12.8%)	1,858 (16.5%)
	70–79	128,182 (25.9%)	8,169 (25.0%)	2,981 (26.4%)
	80–89	146,913 (29.7%)	13,114 (40.2%)	3,879 (34.4%)
	≥90	54,125 (10.9%)	5,594 (17.1%)	1,484 (13.2%)
Male sex		296,707 (59.8%)	18,320 (56.0%)	6,233 (54.9%)
Comorbidity				
Hypertension		264,709 (53.3%)	17,265 (52.8%)	5,808(51.1%)
Diabetes mellitus		122,750 (24.7%)	8,088 (24.7%)	2,742 (24.1%)
Hyperlipidemia		137,506 (27.7%)	6,619 (20.3%)	2,556 (22.5%)
Chronic kidney disease		55,099 (11.1%)	3,944 (12.1%)	1,141(10.0%)
Carlson score		2 (1–3)	3 (2–4)	2 (1–3)
Clinical outcome				
In-hospital mortality		45,741 (9.2%)	4,280 (13.1%)	1,935 (17.0%)
ADL score at discharge		100 (55–100)	65 (5–100)	55 (0–100)
Length of hospital stay, days		13 (7–22)	17 (9–23)	24 (13–41)
Total cost of hospitalization, U.S. dollars		10,351 (5,305–19,260)	8,986 (5,130–16,875)	14,644 (7,983–26,296)
**(B)**				
		**Cerebrovascular disease**		
		**No cardiovascular disease**	**Cardiovascular comorbidity**	**Cardiovascular complication**
Number		281,999	63,647	11,834
Age group, years		73 (64–82)	80 (72–86)	78 (69–85)
	<60	51,770 (18.4%)	3,508 (5.5%)	1,166 (9.9%)
	60–69	61,410 (21.8%)	8,743 (13.8%)	1,916 (16.3%)
	70–79	77,847 (27.7%)	18,432 (29.0%)	3,356 (28.5%)
	80–89	72,601 (25.8%)	24,280 (38.2%)	4,019 (34.2%)
	≥90	17,607 (6.3%)	8,553 (13.5%)	1,303 (11.1%)
Male sex		159,859 (56.7%)	34,697 (54.5%)	6,529 (55.2%)
Comorbidity				
Hypertension		150,755 (53.5%)	33,420 (52.5%)	6,421 (54.3%)
Diabetes mellitus		62,246 (22.1%)	12,845 (20.2%)	3,077 (26.0%)
Hyperlipidemia		68,516 (24.3%)	11,739 (18.4%)	2,922 (24.7%)
Chronic kidney disease		10,805 (3.8%)	3,217 (5.1%)	677 (5.7%)
Carlson score		1 (1–2)	2 (1–3)	2 (1–3)
Clinical outcome				
In-hospital mortality		27,217 (9.7%)	6,691 (10.5%)	1,521 (12.9%)
ADL score at discharge		70 (5–100)	50 (0–100)	50 (0–100)
Length of hospital stay, days		18 (10–32)	21 (12–36)	24 (14–40)
Total cost of hospitalization, U.S.dollars		9,037 (5,245–15,850)	10,465 (6,158–17,434)	12,421 (7,377–21,889)

Values are expressed as numbers (%) or medians (IQR).

ADL, activities of daily living; IQR, inter-quartile range.

Table 2 showed the results of univariate and multivariable multilevel logistic analyses for in-hospital mortality. Of all records, involving cardiovascular disease with the no cerebrovascular disease group as a reference, OR (95% confidence interval) for in-hospital mortality with a cerebrovascular comorbidity and complication was 1.33 (1.28–1.37) and 2.16 (2.05–2.27) in the univariate analysis, 1.15 (1.11–1.19) and 2.02 (1.92–2.13) in Model I, and 1.10 (1.06–1.14) and 2.02 (1.91–2.13) in Model II, respectively. Having a cerebrovascular comorbidity or complication was associated with higher in-hospital mortality in patients hospitalized due to cardiovascular disease. Furthermore, of all records involving cerebrovascular disease with the no cardiovascular disease group as the referent, the OR (95% confidence interval) for in-hospital mortality with a cardiovascular comorbidity and complication was 1.10 (1.06–1.13) and 1.38 (1.31–1.46) in the univariate analysis, 0.96 (0.93–0.99) and 1.26 (1.19–1.33) in Model I, and 0.87 (0.85–0.90) and 1.29 (1.22–1.37) in Model II, respectively. Only the presence of a cardiovascular complication was associated with higher in-hospital mortality in patients hospitalized due to cerebrovascular disease.

**Table 2:** Univariate and multivariable multilevel logistic regression results for in-hospital mortality.

	Univariate	Model I	Model II
**Cardiovascular disease**			
No cerebrovascular disease	1.00	1.00	1.00
Cerebrovascular comorbidity	1.33 (1.28, 1.37)	1.15 (1.11, 1.19)	1.10 (1.06, 1.14)
Cerebrovascular complication	2.16 (2.05, 2.27)	2.02 (1.92, 2.13)	2.02 (1.91, 2.13)
**Cerebrovascular disease**			
No cardiovascular disease	1.00	1.00	1.00
Cardiovascular comorbidity	1.10 (1.06, 1.13)	0.96 (0.93, 0.99)	0.87 (0.85, 0.90)
Cardiovascular complication	1.38 (1.31, 1.46)	1.26 (1.19, 1.33)	1.29 (1.22, 1.37)

Values are expressed as odds ratios (95% confidence intervals).

Model I adjusted for age and sex.

Model II adjusted for age, sex, and comorbidities (hypertension, diabetes mellitus, hyperlipidemia, chronic kidney disease).

Regarding the detailed diagnoses in the comorbidity and complication analysis (S1 Table), CI was the most common comorbidity or complication among all cardiovascular diseases (1.18–3.54%). For AD, CI as a complication was not associated with the in-hospital mortality. On the other hand, there appeared to be differences in incidence and in-hospital mortality among various cerebrovascular diseases (S2 Table). For CI, AF as a comorbidity or complication had the highest incidence (19.20% and 3.20%, respectively). Furthermore, for ICH, HF had the highest incidence for a complication (1.04%). Moreover, AF as a comorbidity and AF as a complication were associated with decreased in-hospital mortality. In addition, for SAH, HF had the highest incidence as both a comorbidity (2.70%) and a complication (1.45%). Moreover, AF as a comorbidity or complication and HF as a complication were associated with lower in-hospital mortality.

### Cardiovascular and cerebrovascular diseases in the rehospitalization analysis

In the rehospitalization analysis, patients with a first hospitalization and rehospitalization were compared (Fig 2). In Table 3, clinical data and outcomes in patients at first hospitalization and rehospitalization due to cardiovascular or cerebrovascular disease are shown by detailed diagnosis. For cardiovascular disease, the higher age and a higher proportion of females were observed at rehospitalization compared with at first hospitalization. Mortality was the highest for AD. HF accounted for the majority of in-hospital mortality. For cerebrovascular disease, the higher age and a higher proportion of females were also observed at rehospitalization compared with at first hospitalization. Mortality was the highest for SAH. CI accounted for the majority of in-hospital mortality.

**Table 3:** Patient characteristics in the rehospitalization analysis.

**(A)**					
	**Cardiovascular disease**				
	**Overall**	**MI**	**HF**	**AF**	**AD**
**First hospitalization**					
Number	319,799	98,462	274,119	84,056	31,013
Age, year	76 (66–85)	71 (62–80)	81 (73–87)	70 (62–78)	72 (63–81)
Male sex	191,226 (59.8%)	70,801 (71.9%)	145,310 (53.0%)	54,594 (65.0%)	17,888 (57.7%)
Comorbidity					
Hypertension	171,377 (53.6%)	58,916 (59.8%)	146,501 (53.4%)	40,171 (47.8%)	19,414 (62.6%)
Diabetes mellitus	85,454 (26.7%)	28,210 (28.7%)	77,580 (28.3%)	14,626 (17.4%)	3,036 (9.8%)
Hyperlipidemia	92,038 (28.8%)	54,792 (55.7%)	56,627 (20.7%)	19,387 (23.1%)	5,859 (18.9%)
Chronic kidney disease	40,692 (12.7%)	6,225 (6.3%)	44,308 (16.2%)	3,937 (4.7%)	1,811 (5.8%)
Mortality rate, %	11.17	12.58	10.59	1.15	19.31
**Rehospitalization**					
Number	32,860	4,789	21,690	5,475	2,352
Age, year	81 (73–87)	77 (69–84)	84 (77–89)	76 (68–83)	76 (67–83)
Male sex	18,458 (56.2%)	3,210 (67.0%)	11,373 (52.4%)	3,401 (62.1%)	1,247 (53.0%)
Comorbidity					
Hypertension	17,066 (51.9%)	2,594 (54.2%)	10,964 (50.6%)	2,902 (53.0%)	1,380 (58.7%)
Diabetes mellitus	8,014 (24.4%)	1,415 (29.6%)	5,688 (26.2%)	1,007 (18.4%)	256 (10.9%)
Hyperlipidemia	6,752 (20.6%)	1,771 (37.0%)	3,651 (16.8%)	1,256 (22.9%)	403 (17.1%)
Chronic kidney disease	3,908 (11.9%)	418 (8.7%)	3,175 (14.6%)	272 (5.0%)	166 (7.1%)
Mortality rate, %	14.77	20.38	15.15	2.85	24.36
**(B)**					
	**Cerebrovascular disease**				
	**Total**	**CI**	**ICH**	**SAH**	
**First hospitalization**					
Number	321,499	240,419	78,029	26,161	
Age, year	75 (65–83)	76 (67–84)	72 (62–82)	66 (53–77)	
Male sex	179,945 (56.0%)	140,401 (58.4%)	43,784 (56.1%)	8,670 (33.1%)	
Comorbidity					
Hypertension	170,604 (53.1%)	122,485 (51.0%)	49,469 (63.4%)	12,896 (49.3%)	
Diabetes mellitus	70,781 (22.0%)	61,332 (25.5%)	12,180 (15.6%)	2,228 (8.5%)	
Hyperlipidemia	75,795 (23.6%)	68,291 (28.4%)	9,372 (12.0%)	3,222 (12.3%)	
Chronic kidney disease	13,422 (4.2%)	10,378 (4.3%)	3,595 (4.6%)	407 (1.6%)	
Mortality rate, %	9.79	5.54	16.82	28.16	
**Rehospitalization**					
Number	69,576	60,839	7,691	1,438	
Age, year	80 (72–86)	80 (73–87)	78 (69–84)	73 (62–83)	
Male sex	37,731 (54.2%)	32,901 (54.1%)	4,576 (59.5%)	477 (33.2%)	
Comorbidity					
Hypertension	36,347 (52.2%)	30,720 (50.5%)	5,107 (66.4%)	752 (52.3%)	
Diabetes mellitus	14,224 (20.4%)	12,656 (20.8%)	1,472 (19.1%)	178 (12.4%)	
Hyperlipidemia	12,839 (18.5%)	11,713 (19.3%)	1,013 (13.2%)	176 (12.2%)	
Chronic kidney disease	3,723 (5.4%)	3,171 (5.2%)	508 (6.6%)	60 (4.2%)	
Mortality rate, %	11.42	9.79	19.46	35.95	

Values are expressed as numbers (%) or medians (interquartile range).

AD, aortic dissection; AF, atrial fibrillation; CI, cerebral infarction; HF, heart failure; ICH, intracerebral hemorrhage; MI, myocardial infarction; SAH, subarachnoid hemorrhage.

**Fig 3:**
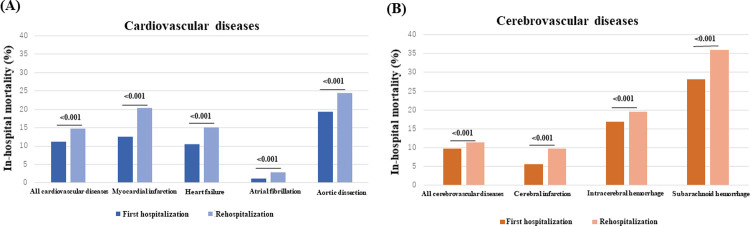
Comparison of in-hospital mortality between first hospitalization and rehospitalization due to cardiovascular disease (A) or cerebrovascular disease (B).

For all cardiovascular diseases, in-hospital mortality rate during rehospitalization was significantly higher than that during first hospitalization (Fig 3A). In the detailed diagnosis analysis, mortality rate was higher for rehospitalization. Similarly, for all cerebrovascular diseases, mortality rate during rehospitalization was significantly higher than mortality rate during first hospitalization, which was also true in the detailed diagnosis analysis (Fig 3B).

## Discussion

We analyzed the frequency and clinical characteristics of cardiovascular and cerebrovascular diseases as comorbidities and complications in order to clarify associations between these two types of diseases. We also investigated mortality rate during rehospitalization for each diagnosis and compared it to mortality rate during the first hospitalization. To the best of our knowledge, our study was the first to comprehensively analyze linkages between cardiovascular and cerebrovascular diseases using a large-scale DPC dataset.

### Relationship between cardiovascular and cerebrovascular diseases as comorbidities and complications

In the present study, the proportion of patients hospitalized due to cerebrovascular disease who had a cardiovascular disease as a comorbidity or complication was higher than the proportion of patients hospitalized due to cardiovascular disease who had a cerebrovascular disease as a comorbidity or complication. This finding might suggest that cerebrovascular disease often develops as a result of cardiovascular disease and that the acute phase of cerebrovascular disease is more unstable and prone to the development of cardiovascular disease [12]. In both conditions, comorbidity or complication status was associated with older age, higher in-hospital mortality rate, longer LOS, decreased ability to perform ADLs at discharge, and higher total cost of hospitalization. However, in patients hospitalized due to cardiovascular disease, the differences were more pronounced. Of note, incident cerebrovascular disease (complication) at the time of cardiovascular hospitalization was associated with the poorest prognosis, suggesting that it is a situation that merits close attention. Multivariable analysis for in-hospital mortality also supported the findings.

The etiology of CI is classified as atherothrombotic, cardioembolic, or lacunar based on the mechanism. There is a strong link between CI and cardiovascular diseases based on atherosclerosis and thromboembolism [13]. Thus, CI was the most common comorbidity and complication among patients hospitalized for any cardiovascular disease. Among cardiovascular diseases, AD tended to be a comorbidity or complication of cerebrovascular disease most frequently, which might be associated with abrupt and substantial hemodynamic changes in AD. A relationship between MI and stroke has been frequently reported [14]. Previous studies have shown an association between cerebrovascular disease and MI. Of 9,180 patients admitted for ICH, 64.9% of discharged patients who had in-hospital MI as a complication died, compared with 35.8% in the entire cohort [15]. A meta-analysis that included 39 studies and 65,996 patients showed that patients admitted for cerebrovascular disease have a relatively high risk of mortality rate due to MI [16]. In the present analysis, MI was not a common complication, but MI as a complication in patients with any cerebrovascular disease was associated with higher in-hospital mortality (OR, 3.30–9.04) in the multivariable analysis. Cautious care for incident MI is required during the management of patients hospitalized due to cerebrovascular disease. Among patients hospitalized for CI, AF was the most frequent comorbidity and complication. A registry that compared patients with CI based on whether they had AF as a comorbidity showed that those with AF had a higher risk of mortality; their 1-year mortality rate was 30.5% [17, 18]. In the present study, CI with AF as a comorbidity or complication was associated with higher in-hospital mortality (OR, 1.63 and 1.23, respectively) in the multivariable analysis. Furthermore, a recent guideline showed that AF remains a common high-risk condition for the onset of a second stroke, which was consistent with our results [19].

### Comparison of first hospitalization and rehospitalization due to cardiovascular or cerebrovascular disease

Mortality during rehospitalization was significantly higher than mortality during first hospitalization for both cardiovascular and cerebrovascular disease, regardless of etiology. However, for cerebrovascular disease, there were small differences in mortality between the first hospitalization and rehospitalization, especially for ICH. Although the precise reasons for the differences are unknown, ICH might not be strongly associated with cardiovascular diseases in general, but ICH might be associated with some specific cardiovascular diseases. Regarding incidence and the number of patients, prevention of incident HF is important in patients with cerebrovascular disease. It is important to prevent incident CI in patients with cardiovascular disease. In the present analysis, in-hospital mortality rate from MI and AF was substantially higher for rehospitalization than for first hospitalization. In contrast, in-hospital mortality for CI was substantially higher for rehospitalization than for the first hospitalization. Based these differences in mortality rate, prevention of incident MI and AF is important in patients with cerebrovascular disease. Similarly, it is important to prevent incident CI in patients with cardiovascular disease.

### Clinical implications

The current guidelines regarding secondary prevention of cardiovascular or cerebrovascular disease recommend strict control and intensive management of cardiovascular risk factors in “very high-risk” patients [20, 21]. All patients with atherosclerotic vascular disease are classified into a very high-risk group; however, the interplay between cardiovascular and cerebrovascular diseases is not considered. The present results suggest that more intensive prevention of progression and management of both cardiovascular and cerebrovascular disease are crucial. In addition, the present findings might be used by other researchers to design clinical trials, epidemiologic studies, and quality outcomes research studies regarding the associations between cardiovascular and cerebrovascular diseases. Such studies might identify more practical and effective prevention and therapeutic strategies to manage cardiovascular and cerebrovascular diseases.

### Study limitations

This study had several limitations. First, since the present analysis was based on the DPC dataset, there are no data about laboratory results and imaging examinations, detailed etiology, and cause of death. Thus, more details about factors associated with mortality or rehospitalization could not be determined. Second, each institution was required to provide their DPC information annually and JROAD-DPC did not necessarily include consecutive DPC datasets. Therefore, these results might underestimate the probability of rehospitalization across the 2 years. Third, since the DPC dataset was a limited annual dataset in 2016 and 2017, the first hospitalization in this study does not necessarily mean the patient’s first-ever episode of disease during their lifetime. Instead, it reflects first-ever episode of disease during the year of interest. Finally, even though mortality and incidence of cerebrovascular disease in Japan are similar to those in Western countries, the mortality and incidence rates of cardiovascular disease in Japan are still lower than those in Western countries [22]. Thus, the present findings might be underestimated compared with those in Western countries. Further international or comparative studies are necessary.

## Conclusions

Substantial associations were observed between cardiovascular and cerebrovascular disease in a Japanese nationwide claims-based dataset. Of note, patients hospitalized with cerebrovascular disease frequently had cardiovascular disease as a comorbidity or complication. These associations had a significant impact on clinical outcomes such as in-hospital mortality, LOS, and total medical costs during rehospitalization. In patients with cardiovascular or cerebrovascular disease, more intensive prevention and management of the other disease are crucial. Therefore, the significant association between cardiovascular and cerebrovascular disease might be an important theme for future research.

## Supporting information

S1 Table Univariate and multivariable analysis of comorbidities and complications for in-hospital mortality in patients with cardiovascular disease. Values are expressed as odds ratios (95% confidence interval). AD, aortic dissection; AF, atrial fibrillation; CI, cerebral infarction; HF, heart failure; ICH, intracerebral hemorrhage; MI, myocardial infarction; SAH, subarachnoid hemorrhage. Model I adjusted for age and sex. Model II adjusted for age, sex, and comorbidities (hypertension, diabetes mellitus, hyperlipidemia, chronic kidney disease).
(DOCX)

S2 Table Univariate and multivariable analysis of comorbidities and complications for in-hospital mortality in patients with cerebrovascular disease. Values are expressed as odds ratios (95% confidence interval). AD, aortic dissection; AF, atrial fibrillation; CI, cerebral infarction; HF, heart failure; ICH, intracerebral hemorrhage; MI, myocardial infarction; SAH, subarachnoid hemorrhage. Model I adjusted for age and sex. Model II adjusted for age, sex, and comorbidities (hypertension, diabetes mellitus, hyperlipidemia, chronic kidney disease).
(DOCX)
