# Supplementary material for: Associations among cardiovascular and cerebrovascular diseases: Analysis of the nationwide claims-based JROAD-DPC dataset
Source: PLoS One. 2022 Mar 11;17(3):e0264390. doi: 10.1371/journal.pone.0264390 (PMC8916648; doi:10.1371/journal.pone.0264390)
Supplement: S1 Table — Values are expressed as odds ratios (95% confidence interval). AD, aortic dissection; AF, atrial fibrillation; CI, cerebral infarction; HF, heart failure; ICH, intracerebral hemorrhage; MI, myocardial infarction; SAH, subarachnoid hemorrhage. Model I adjusted for age and sex. Model II adjusted for age, sex, and comorbidities (hypertension, diabetes mellitus, hyperlipidemia, chronic kidney disease). (DOCX) [file pone.0264390.s001.docx]

|  | **Incidence rate** | **Univariate** | **Model I** | **Model II** |
| --- | --- | --- | --- | --- |
| **MI** |  |  |  |  |
| **Comorbidity** |  |  |  |  |
| CI | 1.18 | 1.53 (1.31, 1.79) | 1.24 (1.06, 1.46) | 0.91 (0.77, 1.09) |
| ICH | 0.27 | 8.95 (6.91, 11.61) | 9.32 (7.07, 12.28) | 4.31 (3.18, 5.84) |
| SAH | 0.06 | 7.89 (4.59, 13.56) | 8.27 (4.63, 14.75) | 4.98 (2.49, 9.95) |
| **Complication** |  |  |  |  |
| CI | 1.37 | 1.91 (1.67, 2.19) | 1.67 (1.45, 1.92) | 1.49 (1.28, 1.75) |
| ICH | 0.19 | 5.57 (4.11, 7.54) | 5.40 (3.92, 7.44) | 4.43 (3.06, 6.39) |
| SAH | 0.04 | 5.23 (2.74, 9.98) | 6.30 (3.21, 12.36) | 3.74 (1.77, 7.90) |
| **HF** |  |  |  |  |
| **Comorbidity** |  |  |  |  |
| CI | 2.00 | 1.18 (1.09, 1.28) | 1.13 (1.04, 1.22) | 1.09 (1.01, 1.18) |
| ICH | 0.15 | 4.68 (3.84, 5.70) | 4.58 (3.74, 5.61) | 3.70 (3.00, 4.57) |
| SAH | 0.03 | 2.63 (1.54, 4.50) | 2.80 (1.62, 4.83) | 2.31 (1.33, 4.01) |
| **Complication** |  |  |  |  |
| CI | 1.57 | 2.39 (2.22, 2.57) | 2.34 (2.17, 2.52) | 2.36 (2.19, 2.55) |
| ICH | 0.18 | 4.80 (3.99,5 .78) | 4.89 (4.04, 5.91) | 4.88 (4.00, 5.95) |
| SAH | 0.02 | 11.03 (6.32, 19.26) | 11.63 (6.53, 20.71) | 11.00 (6.06, 19.97) |
| **AF** |  |  |  |  |
| **Comorbidity** |  |  |  |  |
| CI | 1.58 | 1.43 (0.98, 2.08) | 1.22 (0.84, 1.78) | 1.21 (0.83, 1.77) |
| ICH | 0.11 | 2.16 (0.76, 6.15) | 1.62 (0.56, 4.72) | 1.38 (0.46, 4.09) |
| SAH | 0.02 | 5.97 (0.73, 49.07) | 10.17 (1.24, 83.67) | 7.48 (0.85, 66.00) |
| **Complication** |  |  |  |  |
| CI | 3.06 | 5.83 (4.86, 6.99) | 3.87 (3.23, 4.64) | 3.99 (3.32, 4.80) |
| ICH | 0.16 | 14.49 (8.71, 24.12) | 9.57 (5.70, 16.08) | 9.98 (5.86, 16.97) |
| SAH | 0.02 | 28.90 (9.00, 92.80) | 36.34 (11.15, 118.47) | 43.22 (12.70, 147.13) |
| **AD** |  |  |  |  |
| **Comorbidity** |  |  |  |  |
| CI | 3.00 | 1.37 (1.16,1.61) | 1.34 (1.13, 1.59) | 1.13 (0.94, 1.36) |
| ICH | 0.63 | 12.18 (8.38, 17.70) | 11.63 (7.93, 17.07) | 7.01 (4.49, 10.93) |
| SAH | 0.12 | 11.29 (5.24, 24.29) | 12.79 (5.77, 28.33) | 8.42 (3.12, 22.72) |
| **Complication** |  |  |  |  |
| CI | 3.54 | 0.93 (0.79, 1.10) | 0.98 (0.82, 1.16) | 0.91 (0.76, 1.10) |
| ICH | 0.29 | 3.98 (2.52, 6.27) | 4.86 (3.03, 7.79) | 4.38 (2.57, 7.44) |
| SAH | 0.05 | 5.23 (1.77, 15.41) | 5.92 (2.01, 17.43) | 3.23 (0.99, 10.46) |
