# Supplementary material for: Associations among cardiovascular and cerebrovascular diseases: Analysis of the nationwide claims-based JROAD-DPC dataset
Source: PLoS One. 2022 Mar 11;17(3):e0264390. doi: 10.1371/journal.pone.0264390 (PMC8916648; doi:10.1371/journal.pone.0264390)
Supplement: S2 Table — Values are expressed as odds ratios (95% confidence interval). AD, aortic dissection; AF, atrial fibrillation; CI, cerebral infarction; HF, heart failure; ICH, intracerebral hemorrhage; MI, myocardial infarction; SAH, subarachnoid hemorrhage. Model I adjusted for age and sex. Model II adjusted for age, sex, and comorbidities (hypertension, diabetes mellitus, hyperlipidemia, chronic kidney disease). (DOCX) [file pone.0264390.s002.docx]

|  | **Incidence rate** | **Univariate** | **Model I** | **Model II** |
| --- | --- | --- | --- | --- |
| **CI** |  |  |  |  |
| **Comorbidity** |  |  |  |  |
| MI | 0.40 | 3.67 (2.96, 4.55) | 3.36 (2.70, 4.19) | 2.99 (2.39, 3.74) |
| HF | 9.08 | 2.78 (2.63, 2.93) | 2.07 (1.96, 2.19) | 1.87 (1.77, 1.98) |
| AF | 19.20 | 2.27 (2.17, 2.36) | 1.79 (1.71, 1.87) | 1.63 (1.56, 1.70) |
| AD | 0.25 | 3.17 (2.38, 4.22) | 3.47 (2.59, 4.65) | 3.19 (2.37, 4.30) |
| **Complication** |  |  |  |  |
| MI | 0.33 | 8.24 (6.80, 9.99) | 8.20 (6.74, 9.99) | 9.04 (7.36, 11.11) |
| HF | 1.88 | 4.80 (4.35, 5.29) | 3.97 (3.60, 4.38) | 4.01 (3.62, 4.44) |
| AF | 3.20 | 1.45 (1.30, 1.63) | 1.24 (1.10, 1.38) | 1.23 (1.10, 1.38) |
| AD | 0.16 | 8.74 (6.68, 11.44) | 9.23 (6.99, 12.19) | 8.74 (6.56, 11.64) |
| **ICH** |  |  |  |  |
| **Comorbidity** |  |  |  |  |
| MI | 0.37 | 3.24 (2.53, 4.14) | 3.00 (2.34, 3.85) | 2.62 (2.02, 3.41) |
| HF | 4.00 | 1.12 (1.02, 1.23) | 0.98 (0.89, 1.08) | 0.99 (0.89, 1.09) |
| AF | 5.42 | 0.97 (0.90, 1.06) | 0.80 (0.73, 0.87) | 0.84 (0.77, 0.92) |
| AD | 0.19 | 3.50 (2.48, 4.94) | 3.38 (2.38, 4.79) | 3.32 (2.30, 4.79) |
| **Complication** |  |  |  |  |
| MI | 0.21 | 3.62 (2.61, 5.01) | 3.74 (2.68, 5.20) | 4.32 (3.05, 6.11) |
| HF | 1.04 | 1.82 (1.55, 2.15) | 1.65 (1.40, 1.95) | 1.93 (1.62, 2.30) |
| AF | 0.77 | 0.73 (0.57, 0.94) | 0.62 (0.49, 0.80) | 0.71 (0.55, 0.91) |
| AD | 0.06 | 2.35 (1.22, 4.52) | 2.19 (1.13, 4.23) | 2.42 (1.21, 4.82) |
| **SAH** |  |  |  |  |
| **Comorbidity** |  |  |  |  |
| MI | 0.62 | 7.04 (4.86, 10.19) | 7.11 (4.84,1 0.44) | 5.94 (3.98, 8.88) |
| HF | 2.70 | 1.31 (1.11, 1.55) | 1.05 (0.88, 1.25) | 1.11 (0.92, 1.33) |
| AF | 1.87 | 0.77 (0.62, 0.96) | 0.55 (0.44, 0.69) | 0.59 (0.47, 0.75) |
| AD | 0.34 | 5.01 (3.12, 8.01) | 6.32 (3.83, 10.42) | 5.31 (3.12, 9.03) |
| **Complication** |  |  |  |  |
| MI | 0.24 | 3.01 (1.78, 5.09) | 3.15 (1.84, 5.40) | 3.30 (1.89, 5.76) |
| HF | 1.45 | 0.74 (0.58, 0.96) | 0.62 (0.48, 0.80) | 0.70 (0.54, 0.92) |
| AF | 0.74 | 0.45 (0.30, 0.69) | 0.33 (0.22, 0.51) | 0.37 (0.23, 0.57) |
| AD | 0.10 | 2.02 (0.91, 4.49) | 2.34 (1.03, 5.30) | 2.17 (0.93, 5.02) |
